# Real-Time Fluorescence Imaging Using Indocyanine Green to Assess Therapeutic Effects of Near-Infrared Photoimmunotherapy in Tumor Model Mice

**DOI:** 10.1177/1536012120934965

**Published:** 2020-07-01

**Authors:** Adrian Rosenberg, Daiki Fujimura, Ryuhei Okada, Aki Furusawa, Fuyuki Inagaki, Hiroaki Wakiyama, Takuya Kato, Peter L. Choyke, Hisataka Kobayashi

**Affiliations:** 1 Molecular Imaging Program, Center for Cancer Research, National Cancer Institute, National Institutes of Health, Bethesda, MD, USA

**Keywords:** cancer treatment efficacy, new optical instrumentation, cancer imaging, cancer therapy, NIR fluorescence imaging

## Abstract

**Background:**

Near-infrared photoimmunotherapy (NIR-PIT) is a cancer therapy that causes an increase in tumor perfusion, a phenomenon termed the super-enhanced permeability and retention effect. Currently, in vivo treatment efficacy of NIR-PIT is observable days after treatment, but monitoring would be improved by more acute detection of intratumor change. Fluorescence imaging may detect increased tumor perfusion immediately after treatment.

**Methods:**

In the first experiment, athymic nude mouse models bearing unilateral subcutaneous flank tumors were treated with either NIR-PIT or laser therapy only. In the second experiment, mice bearing bilateral flank tumors were treated with NIR-PIT only on the left-sided tumor. In both groups, immediately after treatment, indocyanine green was injected at different doses intravenously, and mice were monitored with the Shimadzu LIGHTVISION fluorescence imaging system for 1 hour.

**Results:**

Tumor-to-background ratio of fluorescence intensity increased over the 60 minutes of monitoring in treated mice but did not vary significantly in control mice. Tumor-to-background ratio was highest in the 1 mg kg^−1^ and 0.3 mg kg^−1^ doses. In mice with bilateral tumors, tumor-to-untreated tumor ratio increased similarly.

**Conclusions:**

Acute changes in tumor perfusion after NIR-PIT can be detected by real-time fluorescence imaging.

## Introduction

Near-infrared photoimmunotherapy (NIR-PIT) is a cancer therapy that utilizes a monoclonal antibody (mAb) conjugated to a photosensitizing phthalocyanine dye, IRDye 700DX (IR700) to selectively target cancer cells overexpressing the target cell membrane receptor.^1,2^ The mAb-IR700 conjugate is photosensitive and upon exposure to light in the near infrared (NIR) range, it triggers rapid necrosis in cells to which it is bound, with minimal off-target effects.^3,4^

It has been observed that after NIR-PIT, there is an increase in tumor perfusion, a phenomenon known as super-enhanced permeability and retention (SUPR). In contrast to the mild enhanced permeability and retention effect conventionally reported in many solid tumors, SUPR effects are greater in magnitude and are not solely attributable to the overall leakiness of neoplastic vasculature. Rather, the rapid perivascular cell death and accompanying interstitial pressure drop, capillary dilation, and slowing of blood flow combine to promote extravasation of vascular contents into the tumor.^5-8^

These rapid changes have implications for treatment monitoring. Currently, evaluation of treatment efficacy in vivo is limited to observation of gross morphologic change, which does not appear until days after treatment, or through imaging modalities not easily translated to real-time monitoring in humans, such as optical coherence tomography^9^ and fluorescence lifetime imaging.^3^ Efforts to implement NIR-PIT into clinical practice would be greatly aided by methods that allow for assessments closer to the time of treatment, as this would facilitate decisions regarding the need for additional cycles of therapy or would confirm successful treatment.

One potential method involves the use of indocyanine green (ICG) to track the SUPR effect and thus visualize acute changes in the tumor immediately after treatment. Indocyanine green, a US Food and Drug Administration (FDA) approved NIR fluorescent dye with an excitation peak of 760 nm and an emission peak of approximately 820 nm,^6,10,11^ is a nontoxic water-soluble imaging agent that undergoes exclusive hepatic metabolism and biliary excretion, with a plasma half-life in humans of 3 to 4 minutes.^12^ It has been used in tissue and retinal angiography, surgical oncology, and graft perfusion studies, among others.^6,13-15^ In humans, intravenously administered ICG primarily circulates in the intravascular space, noncovalently bound to albumin as a 70 kDa complex with a fluorescence intensity approximately 2 orders of magnitude greater than in the unbound form.^16-18^

The SUPR effects induced by NIR-PIT have been shown to cause observable intratumor redistribution of fluorescently tagged serum proteins like albumin. Such changes have been characterized using dynamic ICG fluorescence, in which repeated NIR fluorescent images are recorded for 60 minutes after treatment of murine models bearing subcutaneous tumor.^19^ A new video-based NIR fluorescence imaging system (LIGHTVISION; Shimadzu Corporation) can visualize the movement of ICG throughout the body in real time and can be rapidly translated to clinical practice.

In this study, we aim to utilize ICG and real-time NIR fluorescence to detect NIR-PIT treated tumors in murine models. We hypothesize that the extravasation and entrapment of ICG in the tumor bed will cause observable intratumor retention of ICG on real-time NIR fluorescence imaging.

## Material and Methods

### Cell Lines and Culture

The A431 cell line, which expresses human epidermal growth factor receptor (EGFR), was used for all studies. Cells were grown in Roswell Park Memorial Institute Medium 1640 (Life Technologies) containing 10% fetal bovine serum (Life Technologies), 0.03% l-glutamine, 100 units mL^−1^ penicillin, and 100 mg mL^−1^ streptomycin in 5% CO_2_ at 37 °C.

### Reagents and APC Synthesis

The mAb used for Antibody-Photoabsorber conjugate (APC) synthesis was Panitumumab (AMGEN Inc), a fully humanized immunoglobulin G2 mAb against EGFR, maintained at 4 °C in stock solution. The dye was IRDye 700DX NHS Ester (IR700; C_74_H_96_N_12_Na_4_O_27_S_6_Si_3_, molecular weight: 1954.22), and it was purchased from LI-COR Bioscience. All other chemicals were of reagent grade.

One mg (6.8 nmol) of Panitumumab was incubated with 66.8 µg (34.2 nmol) IR700 (10.0 mmol L^−1^ in dimethyl sulfoxide) in 0.1 mol L^−1^ sodium phosphate buffer (pH 8.6) at room temperature for 1 hour. The mixture was subsequently purified with a Sephadex G50 column (PD-10; GE Healthcare). The protein concentrations were confirmed with Coomassie Plus Protein Assay Kit (Pierce Biotechnology) by measuring light absorption at 595 nm (8453 Value System, Agilent Technologies). The concentration of IR700 was measured by absorption at 689 nm with spectroscopy to confirm the average number of fluorophore molecules conjugated to each panitumumab molecule (approximately 4:1). We abbreviate IR700 conjugated to Panitumumab as Pan-IR700. Pan-IR700 was diluted with phosphate buffered saline (PBS) to achieve final concentrations of 500 µg mL^−1^.

### In Vivo Murine NIR-PIT Experiments

All in vivo procedures were conducted in compliance with the Guide for the Care and Use of Laboratory Animal Resources (1996), US National Research Council, and approved by the National Cancer Institute/National Institutes of Health (NIH) Animal Care and Use Committee. Female homozygote athymic nude mice aged 6 to 8 weeks were purchased from Charles River (National Cancer Institute). During treatment, mice were anesthetized with isoflurane. Two million A431 cells were injected subcutaneously in either the left flank or in the bilateral flanks; 5 to 7 days after injection, mice with long axis tumor diameters between 5 and 9 mm and total tumor volume (as given by the formula 0.5 × length × width × depth) between 50 and 150 mm^3^ were administered 100 µg of Pan-IR700 through tail vein injection. After 24 hours, NIR laser light (690 ± 5 nm) was administered at a dose of 50 J/cm^2^ (Modulight Inc ML7710; Cylindrical Light Diffuser Model: R030).

No more than 30 minutes before treatment, ICG (Adooq Biosciences LLC) was dissolved in 1:4 H_2_O:PBS to yield solutions of 0.25, 0.08, and 0.025 mg mL^−1^. Immediately after NIR-PIT, ICG was injected intravenously through the tail vein at either 1, 0.3, or 0.1 mg kg^−1^ doses. During injection, and at 15, 30, and 60 minutes postinjection, mice were recorded for 30 seconds using Shimadzu LIGHTVISION (peak excitation: 780 nm; peak emission: 830 nm). The camera was placed 50 cm from the recording surface, and detector sensitivity to ICG was set to 8, 12, and 20 during injection of 1, 0.3, and 0.1 mg kg^−1^ doses, respectively. In all postinjection recordings, sensitivity was set to 12, 19, and 20 for 1, 0.3, and 0.1 mg kg^−1^ doses, respectively. Videos were simultaneously recorded using a white-light channel, an 800 nm channel, and a merged overlay. All videos are displayed at a rate of 5 frames per second.

For unilateral tumor models, 5 mice were used per treatment group and 3 mice per control group. Control mice with unilateral tumors received laser irradiation but no APC. In bilateral murine models, 3 mice were used; they all received Pan-IR700, but the right tumor was shielded from laser light with aluminum foil to serve as the internal control. Indocyanine green was delivered at 0.3 mg kg^−1^. Mice were excluded from analysis if they died prematurely from anesthesia or, for bilateral models, if tumors were asymmetrical.

### Fluorescence Confirmation and Histology

Tumors were also imaged with in vivo fluorescence images using a Pearl Imager (LI-COR Biosciences) on the 700 and 800 nm channels. Imaging was performed immediately pre-NIR-PIT to confirm the selective uptake of Pan-IR700 in the tumor (700 nm channel) and to confirm the absence of significant noise at 800 nm. Imaging was also performed approximately 60 minutes post-NIR-PIT and ICG injection to confirm the photobleaching of IR700 in the tumor (700 nm) and the selective tumor uptake of ICG (800 nm) in treatment mice (or absence of uptake in control mice).

In the bilateral tumor models, the mice were killed immediately after the final in vivo fluorescence image, and the bilateral tumors were excised and imaged with the Shimadzu LIGHTVISION camera. Tumors were then harvested in 10% formalin and sent for histologic preparation; 6 µm slice sections were fixed on glass slides with hematoxylin and eosin staining and analyzed.

### Statistical and Image Analysis

All statistical analyses were performed with GraphPad Prism (GraphPad Software). Student’s t-test (unpaired, Welch correction) was used to compare the mean value for tumor-to-background ratio (TBR) of each treatment group against the TBR of the 1 mg kg^−1^ control group in unilateral tumor models. This test was also used for comparison of TBRs detected via Pearl Imager versus Shimadzu LIGHTVISION. Tumor-to-background ratios and treated tumor to untreated tumor ratios were measured with Image J (NIH) software.

## Results

### Real-Time In Vivo Fluorescence After NIR-PIT: Unilateral Tumor Models

**Figure 1.**
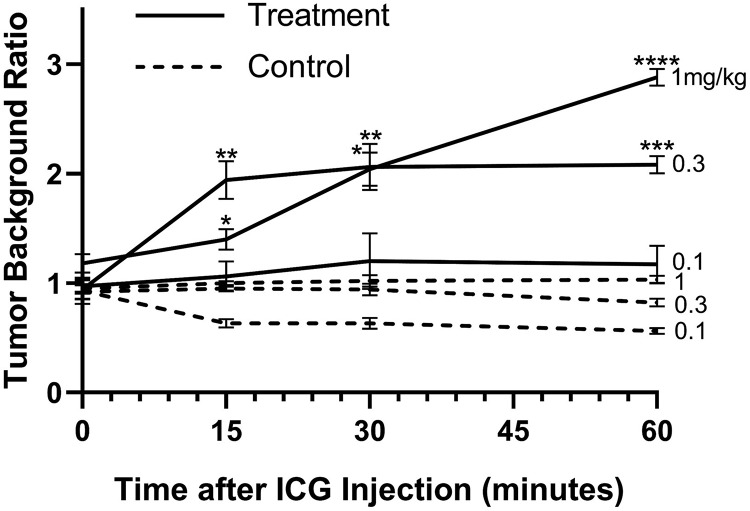
Tumor-to-background ratio of indocyanine green (ICG) fluorescence in flank tumors as detected by Shimadzu LIGHTVISION real-time video monitoring in groups administered varying doses of ICG (1, 0.3, and 0.1 mg kg^−1^, respectively). Background intensity was measured on contralateral flank. Intensities are 10-second averages. Data are means ± SEM (n = 5 for each treatment group; n = 3 for each control group). *P < .05; **P < .01, ***P < .001, ****P < .0001. The *P* values represent difference between the 1 mg kg^−1^ control group and group of interest; Student’s t-test (unpaired). SEM indicates standard error of the mean.

**Figure 2.**
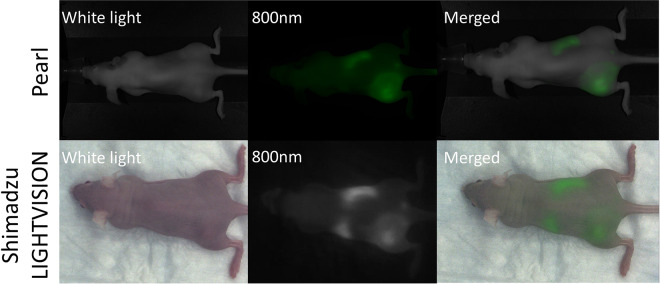
800 nm fluorescence in NIR-PIT-treated mice 60 minutes after intravenous injection with indocyanine green; 1 mg kg^−1^. NIR-PIT indicates near-infrared photoimmunotherapy.

At injection, ICG distributed uniformly through the vasculature at all doses. At 0.3 and 1 mg kg^−1^ doses, selective retention in treated tumors was observable in all treated mice as early as the second time point (15 minutes postinjection), and TBR continued to rise through the 60 minutes of observation (Supplemental Videos 1-3). In control mice, tumor uptake of ICG was not observably higher than background (*P* > .05). At the end of the 60-minute observation period, TBR was significantly elevated in the 1 and 0.3 doses compared to control mice at the same time point (Figure 1; *P* < .001 for 0.3 mg kg^−1^; *P* < .0001 for 1 mg kg^−1^). Confirmatory imaging with in vivo Pearl Imager imaging revealed uptake ratios near identical to that detected by during real-time videos in unilateral tumor models that received 1 mg kg^−1^ (Figure 2) and 0.1 mg kg^−1^, and in bilateral tumor models, which received 0.3 mg kg^−1^ Pearl Imager imaging did show statistically significant higher TBR compared to LIGHTVISION in unilateral models that received 0.3 mg kg^−1^ (mean TBR 3.0 vs 2.1; *P* = .04), though the rate of visual detection of tumors was not different between imaging modalities.

### In Vivo Fluorescence Videography After NIR-PIT: Bilateral Tumor Models

**Figure 3.**
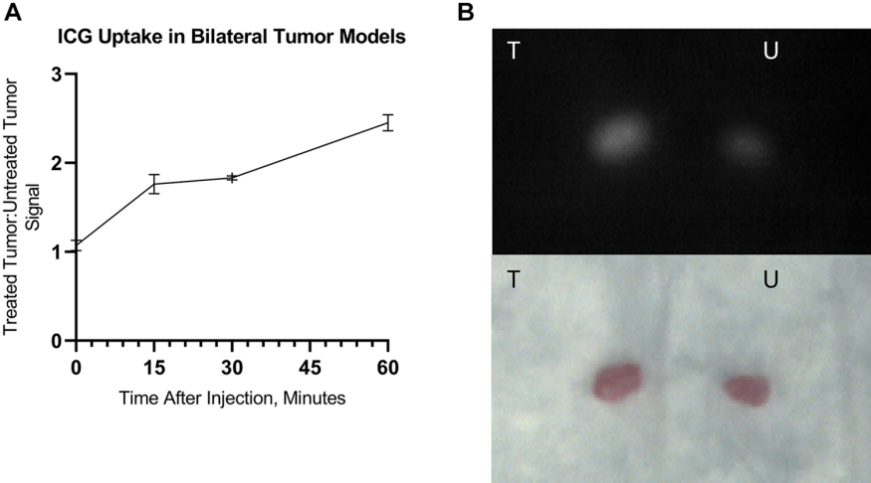
Bilateral flank tumor-bearing mice (A) and their tumors after excision (B). Ratio of indocyanine green (ICG) fluorescence in treated versus untreated tumors over 60 minutes of observation. Injection of ICG was performed immediately after NIR-PIT treatment. Video monitoring was performed with Shimadzu LIGHTVISION camera. ICG dose = 0.3 mg kg^−1^. Intensities are 10-second averages. Data are means ± SEM. N = 3. T = treated; U = untreated. NIR-PIT indicates near-infrared photoimmunotherapy; SEM, standard error of the mean.

Like the unilateral models, ICG distributed evenly through the body of the mice during injection, and upon observation at all subsequent time points, signal was observed in the left (treated) tumors but not in the right (untreated tumors; Supplemental Video 4). Treated-to-untreated tumor ratio was similar to the TBRs observed in unilateral tumor-bearing mice (Figure 3A). Ex vivo tumor imaging showed detectable differences between treated and untreated tumor fluorescence intensity 60 minutes after ICG injection, with preferential ICG uptake in treated tumors (Figure 3B).

### Histologic Analysis

**Figure 4.**
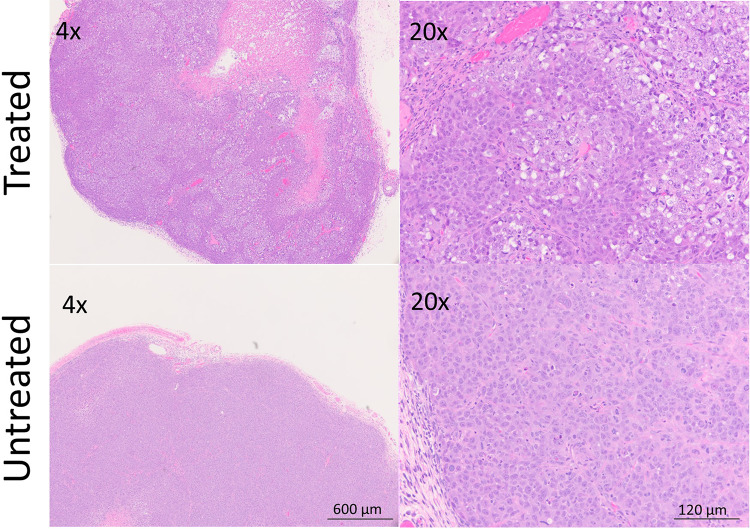
In treated tumor tissue, diffuse necrosis and patchy microhemorrhage are observed. Untreated tumor tissue shows only minimal necrotic changes. Treatment: 50 J/cm^2^. Cell line: A431. Samples preserved in formalin at 1 hour after NIR-PIT. NIR-PIT indicates near-infrared photoimmunotherapy.

Histologic specimen analysis revealed diffuse segments of necrosis within the treated tumors but not in untreated tumors (Figure 4). This correlated well with the pattern of ICG uptake in treated versus untreated tumors.

## Discussion

The advantages of ICG fluorescence imaging have made it a popular agent in medicine: it is noninvasive, portable, safe, relatively inexpensive, exhibits excitation and emission peaks in the NIR range, does not expose subjects to ionizing radiation, and is already FDA approved.^13,20^ Furthermore, ICG is highly localized to the vasculature due to its tendency to bind serum proteins. Even without NIR-PIT treatment, ICG has been shown to preferentially accumulate in tumor tissue compared to less hyperpermeable inflammatory tissue.^10^ Thus, ICG has potential to be an ideal marker of the acute perivascular changes inherent to NIR-PIT.

In this study, we utilized ICG in doses ranging from 0.1 to 1 mg kg^−1^, well below the FDA recommended maximum of 2 mg kg^−1^, and similar to the doses used in humans (most commonly 0.5 mg kg^−1^). As expected, the majority of ICG rapidly accumulated in the liver in all mice, and only in treated tumors did an observable proportion remain trapped in the tumors. The fluorescence intensity ratios were similar among all the imaging modalities and among all fluorescence sensitivity settings tested in the Shimadzu LIGHTVISION. Indocyanine green doses at 0.1 mg kg^−1^ did not reliably produce different uptake in tumor versus background tissue, likely because a high circulating level of unsaturated albumin diluted any tumor uptake of ICG-bound albumin. At a dose of 0.01 mg kg^−1^, fluorescence was not visibly more intense than background objects in the laboratory at even the highest sensitivity settings.

Previous experiments have shown that NIR-PIT initially destroys perivascular tumor cells but tumor vessels remain intact.^4,9^ Furthermore, the impaired venous and lymphatic drainage in tumors, coupled with the rapid decrease in interstitial pressure and dilation of capillaries after NIR-PIT, promotes extravasation of vascular contents into the tumor interstitium after NIR-PIT.^21^ This is the basis for the SUPR effect, which peaks within 6 hours of treatment and returns to near baseline by 24 hours.^22^ The SUPR effects allow residual APC in the circulation to penetrate further into tumor tissue and enhanced delivery of nanodrugs after therapy. This effect is profound resulting in up to 24-fold increase delivery of nanodrugs into the tumor compared to nontreated tumors.^4,7,8,22^ Therefore, the SUPR effect has favorable implications for repeated light exposures and fractionated administration of combination therapies. Here we show that it can also be useful for monitoring the selective tumor uptake of the albumin-bound fluorescent dye, ICG.

While this study shows reliable detection of ICG, demonstrating TBRs of approximately 2:1 and 3:1 in optimal doses, it is worth noting that other studies of non-NIR-PIT treated tumors have shown TBRs in mice as high as 8:1 when higher doses (up to 8 mg kg^−1^) and longer periods of observation (12-72 hours) were employed.^5^

Where real-time ICG imaging shows particular potential is in the intraoperative setting, in which confirmation of adequate treatment can inform repeated light dosing of NIR-PIT without the need to reoperate, making it especially useful for deeper tumors. While there are already some reported intraoperative clinical uses of real-time ICG fluorescence for non-NIR-PIT applications (such as sentinel lymph node mapping,^23^ hemangioblastoma resection,^24^ confirmation of ischemia during partial nephrectomy,^25^ and detection of kidney allograft perfusion defects^12^), our study broadens the scope of possible uses for ICG fluorescence videography to include real-time monitoring of NIR-PIT treatment effects.

Limitations of this study pertain mostly to generalizability to tumors of different size and age. Tumors were chosen within a limited size and date range because of a need for appropriate vascular maturation and patency. Younger and smaller tumors were less likely to exhibit vasculature mature enough to allow for SUPR effects after treatment. Larger and older tumors were more likely to show central necrosis, leading to limited ICG uptake, often localized to the rim around the tumor. In order to perform imaging procedure under the consistent condition, we employed a subcutaneous xenograft tumor model at a fixed location. However, if we could use an orthotopic mouse model of cancer, it would be more realistic to simulate clinical setting.^26-28^

## Conclusions

For subcutaneous tumor models, in vivo ICG NIR fluorescent video imaging allows assessment of treatment efficacy before morphologic changes become evident. Indocyanine green is a sensitive marker of the acute vascular changes induced by NIR-PIT.
